# Daytime Exposure to Short Wavelength-Enriched Light Improves Cognitive Performance in Sleep-Restricted College-Aged Adults

**DOI:** 10.3389/fneur.2021.624217

**Published:** 2021-02-22

**Authors:** Leilah K. Grant, Brianne A. Kent, Matthew D. Mayer, Robert Stickgold, Steven W. Lockley, Shadab A. Rahman

**Affiliations:** ^1^Division of Sleep and Circadian Disorders, Departments of Medicine and Neurology, Brigham and Women's Hospital, Boston, MA, United States; ^2^Division of Sleep Medicine, Harvard Medical School, Boston, MA, United States; ^3^Department of Psychiatry, Beth Israel Deaconess Medical Center, Boston, MA, United States; ^4^Department of Psychiatry, Harvard Medical School, Boston, MA, United States

**Keywords:** light, melanopsin, cognition, learning, melanopic light

## Abstract

We tested the effect of daytime indoor light exposure with varying melanopic strength on cognitive performance in college-aged students who maintained an enforced nightly sleep opportunity of 7 h (i.e., nightly sleep duration no longer than 7 h) for 1 week immediately preceding the day of light exposure. Participants (*n* = 39; mean age ± SD = 24.5 ± 3.2 years; 21 F) were randomized to an 8 h daytime exposure to one of four white light conditions of equal photopic illuminance (~50 lux at eye level in the vertical plane) but different melanopic illuminance [24–45 melanopic-EDI lux (melEDI)] generated by varying correlated color temperatures [3000K (low-melEDI) or 5000K (high-melEDI)] and spectra [conventional or daylight-like]. Accuracy on a 2-min addition task was 5% better in the daylight-like high-melEDI condition (highest melEDI) compared to the conventional low-melEDI condition (lowest melEDI; *p* < 0.01). Performance speed on the motor sequence learning task was 3.2 times faster (*p* < 0.05) during the daylight-like high-melEDI condition compared to the conventional low-melEDI. Subjective sleepiness was 1.5 times lower in the conventional high-melEDI condition compared to the conventional low-melEDI condition, but levels were similar between conventional low- and daylight-like high-melEDI conditions. These results demonstrate that exposure to high-melanopic (short wavelength-enriched) white light improves processing speed, working memory, and procedural learning on a motor sequence task in modestly sleep restricted young adults, and have important implications for optimizing lighting conditions in schools, colleges, and other built environments.

## Introduction

The physiological (non-visual) effects of light in humans range from changes in gene expression (1) to overt behavior (2–4). One of the characteristic non-visual responses to light is the stimulation of alertness and cognitive performance. These responses are mediated by intrinsically photosensitive retinal ganglion cells (ipRGCs), primarily through stimulation of the photopigment melanopsin that is most sensitive to higher intensity ~480-nm light (5). Therefore, high intensity and short wavelength (blue)-enriched light with greater melanopic content is typically more effective in inducing physiologic responses relative to dimmer blue-depleted light with lower melanopic content (6–8).

The spectral sensitivity of non-visual responses to light, including alertness and cognitive performance, has predominantly been examined during evening and nighttime exposures (3, 4, 9–13). Relatively few studies have examined the effects of short-wavelength light on daytime alertness and performance. While comparison of monochromatic or narrow-bandwidth sources of different wavelengths have shown that short-wavelength light preferentially improves daytime alertness and performance (14–16), studies examining the effects of white light with different correlated color temperatures (CCT) have shown mixed results (17–19). As these differences may be a result of methodological inconsistencies, particularly with regard to differences in the duration of exposure (1–16 h) and photopic illuminance, more studies are needed to determine whether blue-enriched white light during the day has a beneficial effect on alertness and cognition.

While there is evidence for the benefits of blue-enriched light on alertness and cognitive performance, there is considerably less understanding of the effect of light spectra on learning and memory. A small number of studies suggest that declarative memory (9, 20), and procedural learning (21) are better under blue-enriched light in the evening, and one study has shown improved verbal memory recall under blue-enriched light during the day (20).

In the current study we aimed to examine the effects of an 8-h daytime light exposure (LE) to one of four polychromatic light emitting diode (LED) light sources with the same photopic (visual) illuminance but different spectral compositions, and therefore different melanopic content estimated by melanopic Equivalent Daylight Illuminance (melEDI) on cognition. It was hypothesized that learning and memory, sleepiness and alertness, and vigilance and concentration would improve with exposure to light with higher melEDI. Additionally, given recent evidence suggesting that daytime alertness is higher with exposure to light with daylight-like spectra compared to light with conventional LED spectra (17), we also compared the effects of conventional and daylight-like spectra within the high- and low-melEDI conditions.

## Materials and Methods

### Participants

Thirty-nine healthy college-aged (18–30 years) participants [21 females; mean age (±SD): 24.5 ± 3.2 years)] were studied in the Intensive Physiological Monitoring (IPM) Unit in the Center for Clinical Investigation (CCI) at Brigham and Women's Hospital. The study was approved by the Partners Human Research Committee (IRB# 2019-P-000900), and participants provided written informed consent prior to study. All participants reported being free from medical and psychological conditions and had a negative Ishihara Color Blindness Test. Participants were either currently enrolled in college or had a college degree. For at least 1 week prior to entering the IPM Unit, participants maintained a consistent sleep/wake schedule that limited time in bed to 7 h (e.g., 23:00–06:00). Participants selected for themselves the 7-h interval for time in bed at the start of the study based on their own preference and schedule, but the same 7-h time in bed was then maintained every night for 7 consecutive nights leading up to the in-lab study. Adherence to the sleep/wake schedule was confirmed with (1) calls to a time- and date-stamped voicemail at bedtime and wake time, and (2) wrist actigraphy (Actiwatch, MiniMitter Company, Inc., Sunriver, OR, USA). The 7-h time in bed was selected based on the average sleep duration of college students being less than 7 h (22, 23). Participants were asked to refrain from use of any prescription or nonprescription medications, supplements, recreational drugs, caffeine, alcohol, or nicotine. Compliance was verified by urine toxicology upon entry to the IPM Unit. At the time of study, approximately half (11/21) of the women were using hormonal contraception (oral birth control *n* = 4; intrauterine device *n* = 5; Nexplanon implant *n* = 2). Of the naturally cycling women not using contraception (*n* = 10), six were in the follicular phase of their menstrual cycle, and they were approximately evenly distributed between the LE conditions.

### Study Protocol

**Figure 1:**
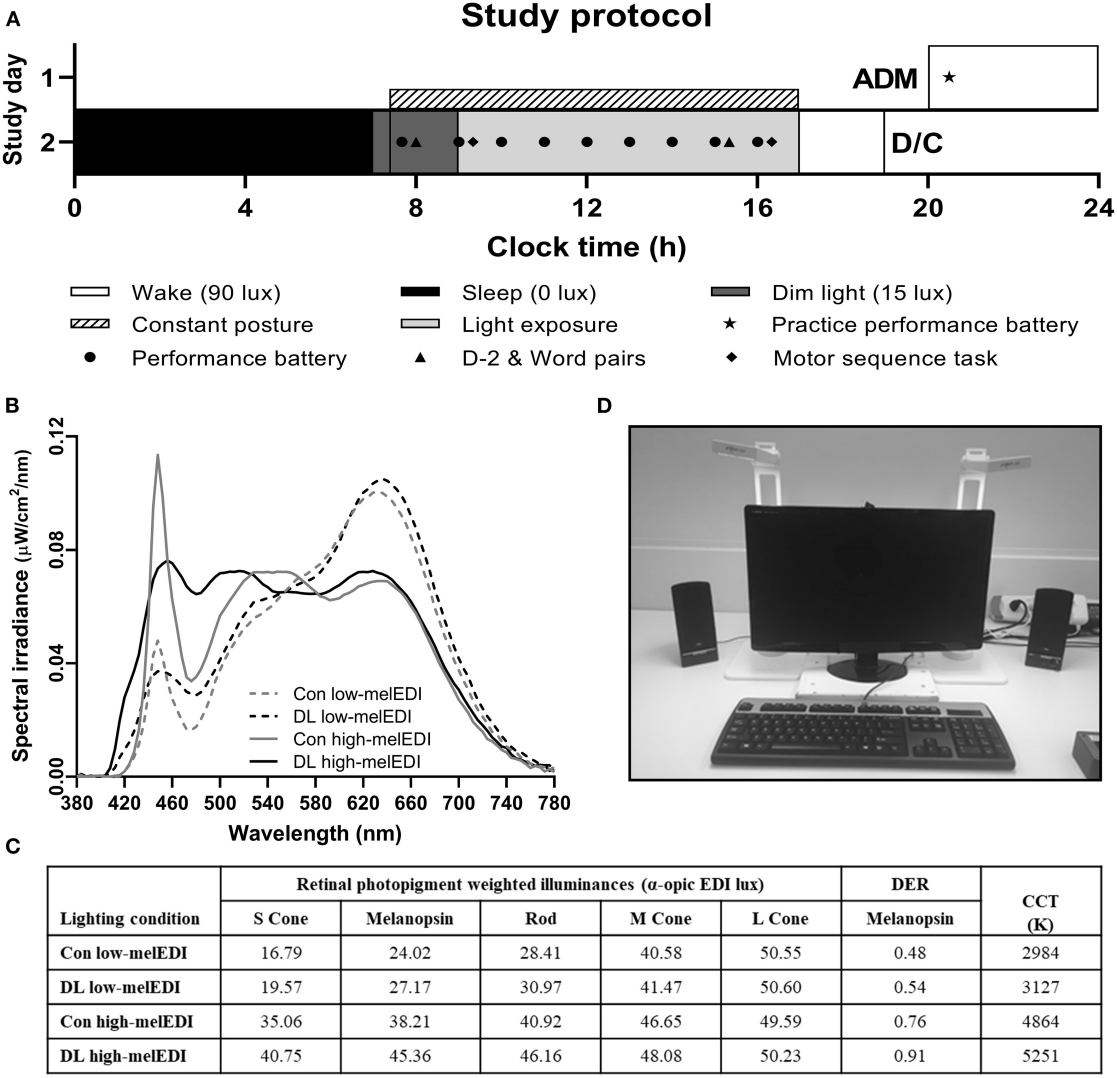
Study protocol and light source spectral characteristics and experimental configuration. The study protocol **(A)** consisted of one night in the laboratory where participants were admitted in the evening and maintained their pre-admit sleep-wake cycle including a 7-h sleep opportunity. On Day 2, participants underwent an 8-h light exposure where they were exposed to ~50 lux of experimental light with differing spectra **(B)** 3000K or 5000K of standard (conventional) or full-spectrum (daylight-like) LED light. During the light exposure, two lamps were configured on either side of the testing station monitor at which participants maintained a constant posture **(C)**. α-opic EDI and DER values for each light source **(D)** were derived from the CIE S 026:2018 Toolbox V1.049. ADM, admission; CCT, correlated color temperature; Con, Conventional; D/C, discharge; DER, daylight (D65) efficacy ratio; DL, Daylight-like; EDI, equivalent daylight (D65) illuminance.

Participants were studied using a 2-day laboratory protocol (Figure 1A) in an environment free of time cues (no access to windows, clocks, watches, live TV, radio, internet, telephones, and newspapers and continually supervised by staff trained not to reveal information about the time of day). Participants were admitted to the Unit ~4 h prior to bedtime and were oriented to their suite following examination by the clinical staff. A 7-h sleep opportunity (time in bed) was scheduled according to the centered average of sleep reported daily for 7 days immediately prior to admission. Upon waking, participants began a constant posture 25 min after wake until the end of the light exposure. Two hours after wake, participants began their experimental LE, which continued for 8 h followed by discharge from the Unit.

### Light Exposure Conditions

On Day 1 (admission), maximum ambient light during scheduled wake was 48 μW/cm^2^ (~150 lux) when measured in the horizontal plane at a height of 187 cm and 23 μW/cm^2^ or (~89 lux) when measured in the vertical plane at a height of 137 cm. During the sleep episode, ambient lighting was switched off (0 lux). Following sleep, maximum ambient light was decreased to 0.05 μW/cm^2^ (~15 lux) in the horizontal plane at a height of 187 cm and 4.8 μW/cm^2^ (~3 lux) when measured in the vertical plane at a height of 137 cm, and maintained at that level until the beginning of the experimental light exposure (Supplemental Table 1). Ambient room lighting was generated using ceiling-mounted 4100K fluorescent lamps (F96T12/41U/HO/EW, 95W; F32T8/ADV841/A, 32W; F25T8/TL841, 25W; Philips Lighting, The Netherlands) with digital ballasts (Hi-Lume 1% and Eco-10 ballasts, Lutron Electronics Co., Inc., Coopersburg, PA) transmitted through a UV-stable filter (Lexan 9030 with prismatic lens, GE Plastics, Pittsfield, MA). Routine illuminance and irradiance measures were conducted using an IL1400 radiometer/powermeter with an SEL-033/Y/W or SEL-033/F/W detector, respectively (International Light, Inc., Newburyport, MA).

Participants were randomized to one of four LE conditions with equal photopic illuminance (~50 lux in the vertical plane at the level of the eye, and ~150 lux in the horizontal plane at the level of the desk) but different melanopic illuminance (25–45 melEDI) generated using LED luminaires with either 5000K (high-melEDI) or 3000K (low-melEDI) CCT, and then further differentiated based on having a conventional or daylight-like spectra (Figures 1B,C). Luminaires were provided by Seoul Semiconductor Co., Ltd. (Ansan-si, Gyeonggi-do, Korea). During the 8-h LE, participants maintained a constant posture while seated at a testing station (Figure 1D), which maintained exposure of ~50 lux in the vertical plane at the level of the eye, and ~150 lux in the horizontal plane at level of the desk (Table 1). All light sources besides the experimental LED lamps remained turned off throughout the 8-h LE. The spectral profiles, CIE α-opic equivalent daylight (D65) illuminance (EDI) and melanopic daylight (D65) efficacy ratio (DER) (24) for each experimental light sources are shown in Figures 1B,C, and for the ambient lighting in Supplemental Table 1. Spectral measurements were conducted using a PR-650 SpectraScan Colorimeter with CR-650 cosine receptor (Photo Research Inc., Chatsworth, CA, USA).

**Table 1:** Participant demographic and photopic illuminance and irradiance measures for each condition^*^.

**Light condition**	**Age,** **years**	**Sex female** **(*n*, %)**	**Bedtime,** **hh:mm**	**Time in bed,** **hh:mm**	**Vertical plane**		**Horizontal plane**	
					**Photopic lux**	**Irradiance,** **μW/cm^**2**^**	**Photopic lux**	**Irradiance,** **μW/cm^**2**^**
Conventional low-melEDI	24.20 (3.35)	5 (55.56)	23:25 (0:44)	07:00 (0:01)	50.13 (0.86)	15.11 (1.54)	150.97 (3.51)	47.81 (3.02)
Daylight-like low-melEDI	24.44 (3.94)	5 (55.56)	23:23 (1:13)	07:04 (0:04)	50.09 (0.71)	17.35 (4.36)	150.03 (2.05)	51.23 (3.09)
Conventional high-melEDI	25.30 (3.47)	5 (50)	23:50 (1:07)	07:07 (0:11)	50.35 (2.11)	18.06 (2.12)	151.19 (4.27)	49.92 (2.24)
Daylight-like high-melEDI	23.56 (1.8)	5 (55.56)	23:58 (0:52)	07:03 (0:02)	50.27 (0.74)	19.03 (2.15)	147.40 (5.55)	52.55 (2.48)

* *Age, bedtime, time in bed, photopic lux, and irradiance are reported as mean ± SD. Sex is reported as number and percent of female participants. Light measurements in the vertical and horizontal planes were taken at the level of the eye and desk, respectively. Time-in-bed was derived from call-ins at bed and wake times*.

### Sleepiness, Wellbeing, Performance, and Learning Assessments

The timing of assessments throughout the light exposure are shown in Figure 1A. The Performance Battery, which included the Psychomotor Vigilance Task [PVT (25)], Addition Task (26), Karolinska Sleepiness Scale [KSS (27)], and Visual Analog Scales [VAS, (26)], was administered once during dim light and then hourly throughout the light exposure. The battery assessed sustained attention (PVT), working memory and processing speed (Addition Task), subjective sleepiness (KSS), and alertness, health and wellbeing (VAS). A brief practice session to familiarize participants with the battery was administered at admit (Figure 1A). Approximately 1 h before lights on, participants completed the d-2 (28) and Word Pairs (29) tasks, which assessed concentration and declarative memory, respectively. These tasks were then repeated 7 h later, 6 h into the light exposure (Figure 1A). Approximately 30 min after lights on, participants completed the Motor Sequence Task [MST, (30)] to assess procedural learning and the Headache and Eye Strain Scale (31). These assessments were repeated 7 h later, 30 min before the end of the LE (Figure 1A). Detailed descriptions of the assessments are provided in the Supplemental Materials and Methods.

### Data Analysis

Data from one female and one male participant (3906V, 3907V; neither reported in Table 1) were excluded from all analyses due to technical failure during the LE. For tests administered hourly during the LE, the median of each outcome measure was calculated across the LE for each individual. For the d-2 test, only the second session, which was performed during the LE, was included in the analysis comparing the different LE conditions. Data from the d-2 task was excluded for one participant (3923V) in the daylight-like low-melEDI condition as they did not adhere to testing instructions. MST task performance was analyzed across the LE such that the average of each trial across both sessions was used in the analysis. Errors on the MST task were square-root transformed (√*x* + √*x*+1) prior to analysis. Headache and Eye Strain Scale responses were dichotomized as None/Mild symptoms (scores of 0 and 1), and Moderate/Severe Symptoms (scores of 3 and 4).

The Shapiro–Wilk test was used to assess normal distribution of the data within each LE condition. Data points that were located more than ±1.5 times the interquartile range were considered outliers and removed from analyses (32). No more than one participant was removed from any LE condition (see Table 2). The effect of LE condition on each outcome variable was assessed by one-way ANOVA or the Kruskal–Wallis test, as appropriate. If a main effect was detected, *post-hoc* tests were performed to compare between the (1) conventional low-melEDI to daylight-like low-melEDI, conventional high-melEDI and daylight-like high-melEDI; and (2) conventional low- and high-melEDI to daylight-like low- and high-melEDI, respectively. Holm-Sidak and Dunn corrections for multiple comparisons were used for the ANOVA and Kruskal–Wallis tests, respectively. Dichotomized data from the Headache and Eye Strain Scale were analyzed using Fisher's Exact test for Session 1 (start of LE) and Session 2 (end of LE). All statistical analyses were conducted in GraphPad Prism (Version 8.4.0 for Windows, GraphPad Software, San Diego CA, USA).

**Table 2:** Mean ± SEM and ANOVA results for each test outcome^*^.

**Test outcome**	**Conventional low-melEDI**		**Daylight-like low-melEDI**		**Conventional high-melEDI**		**Daylight-like high-melEDI**		***F*** **(*p*-value)**
	***M*** **(SEM)**	***N***	***M*** **(SEM)**	***N***	***M*** **(SEM)**	***N***	***M*** **(SEM)**	***N***	
**OBJECTIVE MEASURES**									
PVT reaction time (ms)	270.70 (6.54)	9	256.40 (8.69)	9	264.00 (12.23)	10	266.10 (10.28)	8	0.37 (0.78)
PVT attentional failures	1.33 (0.29)	9	1.11 (0.41)	9	2.25 (0.78)	10	0.81 (0.31)	8	2.84^†^ (0.42)
Additions % correct	93.98 (1.02)	9	94.53 (1.09)	9	96.57 (1.11)	10	98.89 (0.42)	8	13.36^†^ (0.004)
Additions # attempted	25.00 (2.26)	8	30.06 (3.74)	9	23.95 (2.34)	10	24.00 (2.77)	9	1.06 (0.38)
d-2 CP	255.9 (11.66)	9	262.0 (13.13)	8	264.6 (8.51)	10	254.9 (11.54)	9	0.19 (0.91)
d-2 % errors	1.49 (0.26)	9	1.81 (0.42)	8	1.95 (0.34)	10	2.24 (0.50)	9	0.65 (0.59)
MST % change speed	22.32 (9.6)	8	55.08 (14.03)	9	44.83 (7.32)	10	71.16 (9.59)	9	3.68 (0.02)
MST % change errors	54.70 (14.21)	9	3.09 (16.69)	9	15.45 (17.96)	10	−8.33 (14.0)	9	7.20^†^ (0.06)
Word pairs % recall	94.42 (2.57)	9	95.12 (1.60)	9	92.00 (2.25)	10	93.17 (1.70)	9	0.84^†^ (0.84)
**SUBJECTIVE MEASURES**									
KSS	5.00 (0.53)	9	3.72 (0.37)	9	3.35 (0.21)	10	5.22 (0.60)	9	11.30^†^ (0.01)
Sleepy—Alert	60.37 (5.08)	9	76.04 (4.98)	9	71.16 (5.33)	10	63.84 (6.97)	9	4.60^†^ (0.20)
Calm—Stressed	25.48 (4.02)	9	12.89 (3.91)	9	13.50 (3.96)	9	12.84 (3.32)	9	2.66 (0.07)
Sad—Happy	74.43 (5.30)	9	73.93 (5.33)	9	83.63 (4.95)	10	79.96 (3.86)	9	0.92 (0.44)
Healthy—Sick	16.49 (2.48)	9	7.78 (2.50)	9	6.11 (1.42)	9	23.32 (8.05)	9	3.25 (0.03)
Energetic—Exhausted	49.59 (4.52)	9	34.32 (6.22)	9	32.04 (5.14)	10	42.15 (8.02)	9	1.74 (0.18)
Exhausted—Sharp	56.12 (6.52)	9	67.52 (7.05)	9	66.37 (6.38)	10	67.37 (7.65)	9	0.62 (0.60)
Tired—Fresh	54.25 (5.81)	9	71.42 (7.17)	9	66.80 (5.51)	10	62.32 (6.10)	9	1.39 (0.26)
Motivated—Unmotivated	31.64 (2.01)	9	26.74 (6.46)	9	29.57 (5.76)	10	16.97 (4.89)	9	1.50 (0.23)

* M, mean; N, the number of participants in each group that were included in the analysis following removal of outliers; CP, concentration performance;

† *denotes Kruskal–Wallis statistic where data were not normally distributed*.

## Results

There were no significant differences in age, bedtime, or pre-admission time-in-bed between the light condition groups (*p* > 0.05 for all; Table 1). Group mean (±SEM) and statistical test results for objective and subjective measures collected during the LE are presented in Table 2. There were no differences between light conditions in baseline performance for those tests and subjective ratings assessed under dim-light conditions prior to the LE, including the PVT, Addition Task, and d-2, and subjective sleepiness, alertness and general health and wellbeing (*p* > 0.05 for all; Supplemental Table 2).

### Working Memory, Sustained Attention, and Concentration

**Figure 2:**
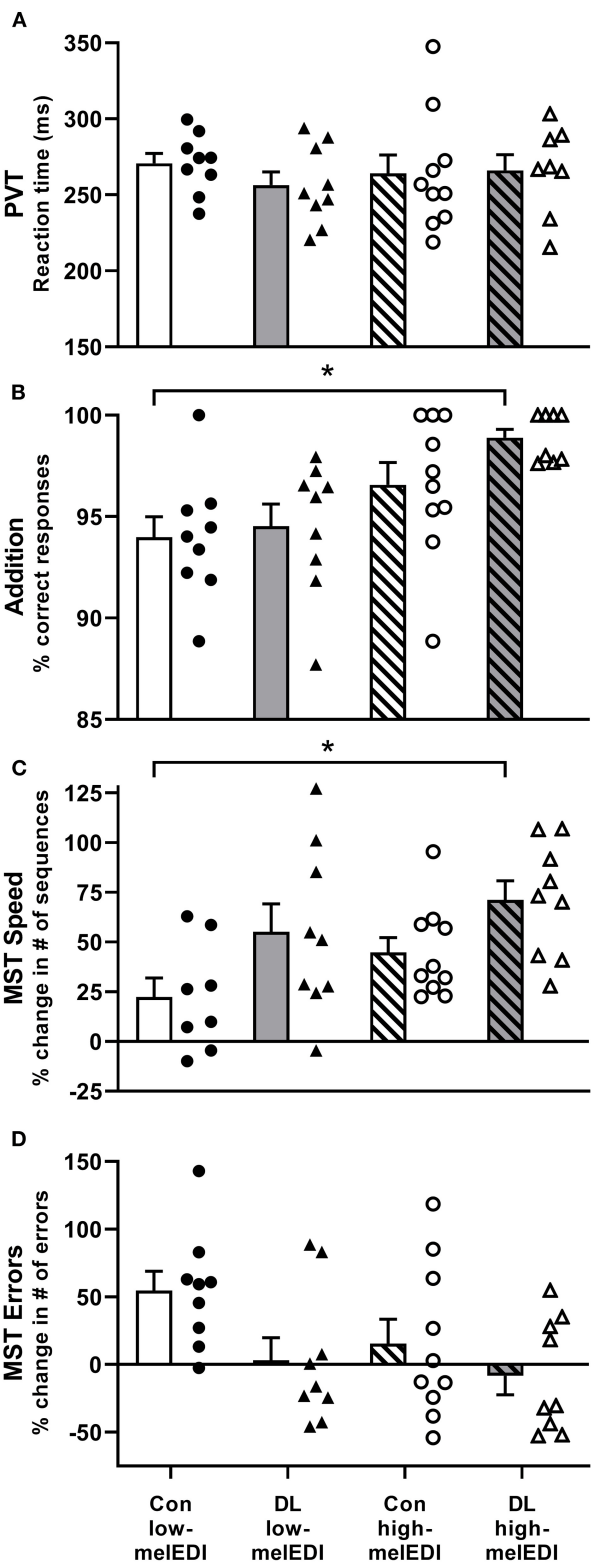
Performance on the PVT, addition task, and motor sequence task for each light exposure condition. Mean ± SEM of PVT reaction time (A), addition task percent correct **(B)**, Motor Sequence Task percent change in the number of correct sequences **(C)**, and Motor Sequence Task percent change in the number of errors **(D)** for each light exposure condition. Individual participant data are shown for conventional low-melEDI (•), daylight-like low-melEDI (▴), conventional high-melEDI (◦), and daylight-like high-melEDI (Δ) light conditions. PVT, psychomotor vigilance task; MST, Motor Sequence Task; Con, Conventional; DL, Daylight-like. *denotes a significant difference between light conditions.

The percentage correct responses on the Addition Task was significantly different between LE conditions (Kruskal–Wallis; *H* = 13.36, *p* < 0.01), such that participants exposed to the daylight-like high-melEDI light performed better than participants exposed to the conventional low-melEDI light (*z* = 3.3, *p* < 0.01). Although there appeared to be a monotonic improvement in the percentage of correct responses with increasing melanopic illuminance, we did not detect a statistically significant difference between the intermediate melEDI conditions (daylight-like low-melEDI and conventional high-melEDI) and the conventional low-melEDI condition (Figure 2B). There were no significant differences between LE conditions in reaction time and attentional failures on the PVT, the number of attempted responses on the Addition task, or accuracy and percentage of errors on the d-2 task (Table 2).

### Procedural Learning and Declarative Memory

Improvement in performance speed across trials (trials 10–12 relative to trial 1) on the MST task was significantly different between the groups (ANOVA; *F* = 3.68, *p* < 0.05; Table 2, Figure 2C). *Post-hoc* analyses showed that improvement in performance speed across trials was significantly greater in the daylight-like high-melEDI condition compared to the conventional low-melEDI condition (*t* = 3.24, *p* < 0.05), but not for the intermediate conditions. Accuracy on the MST task increased with increasing melEDI exposure although this difference only approached statistical significance (*p* = 0.06, Figure 2D). There was no significant effect of light condition on percent recall on the word pairs task (Table 2).

### Subjective Sleepiness, Health, and Wellbeing

**Figure 3:**
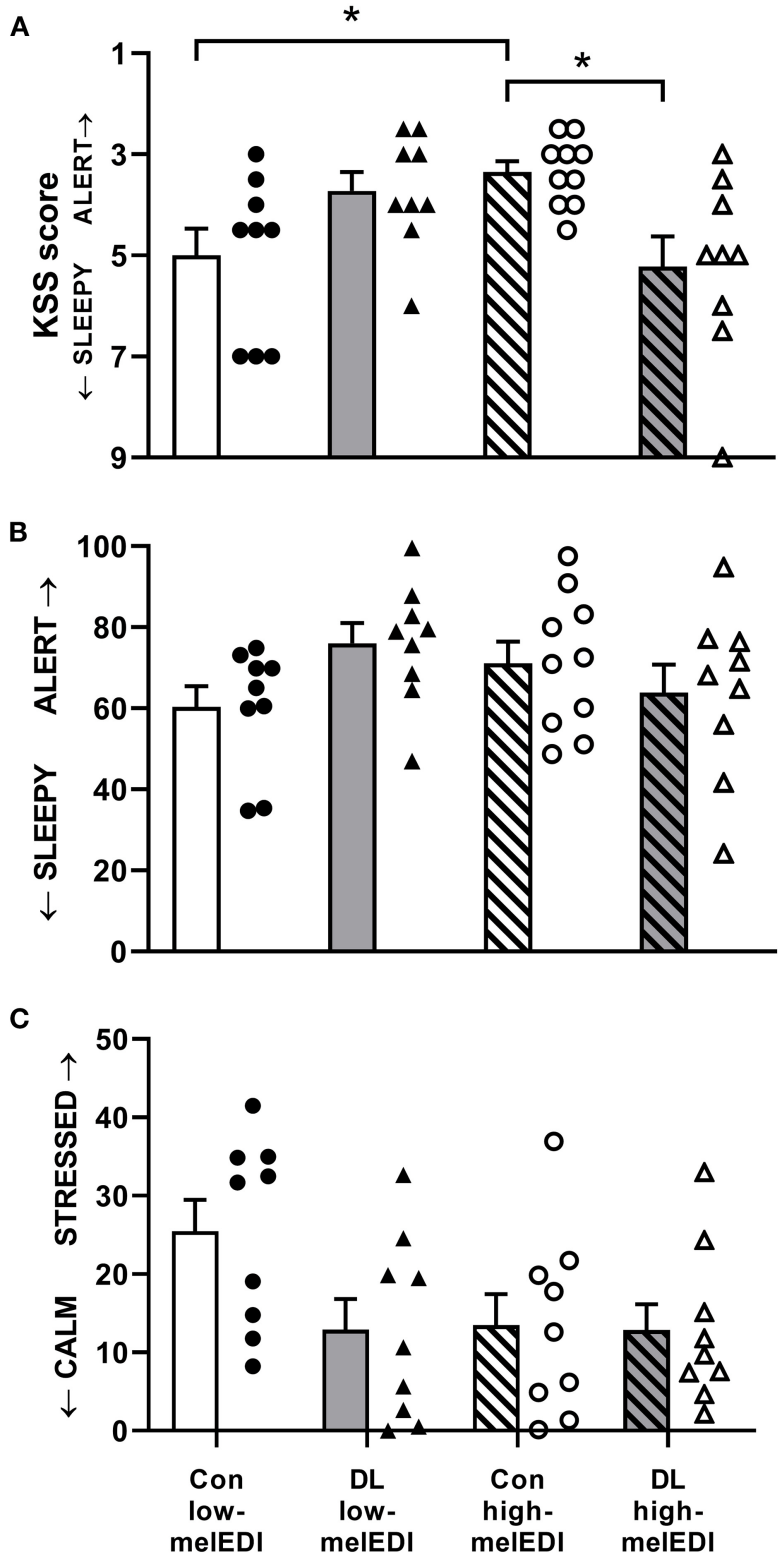
Subjective sleepiness, alertness, and stress for each light exposure condition. Mean ± SEM of KSS sleepiness scores **(A)**, VAS alertness ratings **(B)**, and VAS stress ratings **(C)**. Individual participant data are shown for conventional low-melEDI (•), daylight-like high-melEDI (▴), conventional high-melEDI (◦), and daylight-like melEDI (Δ) light conditions. KSS, Karolinska sleepiness scale; Con, Conventional; DL, Daylight-like. *denotes a significant difference between light conditions.

There was a significant effect of light condition on KSS scores (Kruskal–Wallis; *H* = 11.3, *p* < 0.05; Table 2; Figure 3A). Participants in the conventional high-melEDI condition had lower KSS scores, indicating lower subjective sleepiness, compared to participants in the conventional low-melEDI condition (*z* = 2.56, *p* < 0.05). Conversely, participants in the daylight-like high-melEDI condition reported significantly greater subjective sleepiness than participants in the conventional high-melEDI condition (*z* = 2.75, *p* = 0.02). Additional *post-hoc* contrasts were not statistically significant (Figure 3A). In contrast to KSS ratings of sleepiness, the VAS for “sleepy-alert” was not different between conditions (Figure 3B).

There was a significant effect of LE condition on VAS ratings for “healthy-sick” (ANOVA; *F* = 3.25, *p* < 0.05). Participants in the daylight-like high-melEDI condition reported feeling significantly more sick than participants in the conventional low-melEDI condition (*t* = 2.74, *p* < 0.05); however, one participant in the daylight-like high-melEDI group, while not a statistical outlier, rated themselves consistently as more sick compared to other participants, and was clinically documented as displaying “Common Cold” symptoms during the LE, which were absent at admission to the laboratory. Additional sensitivity analysis with removal of this participant from the “healthy-sick” scale data showed that the *post-hoc* comparison was no longer significant (*p* = 0.1). VAS ratings on the “calm-stressed” scale trended toward being lower in all LE conditions compared to the conventional low-melEDI condition (Figure 3C) although this difference did not reach statistical significance (*p* = 0.07). There was no statistical difference between the LE conditions on any other VAS scales.

### Headache and Eye Strain

There was no difference between any of the lighting conditions in irritability, headache, eye strain, eye discomfort, eye fatigue, or blurred vision assessed during the light exposure (*p* > 0.3 for all; Supplemental Table 3).

## Discussion

Our results show that compared to being exposed to lower melEDI light, exposure to short-wavelength enriched higher melEDI light during the daytime is associated with significantly less sleepiness, better working memory, processing speed, and procedural learning in moderately sleep-restricted college-aged adults. We did not, however, find a statistically significant difference between lighting conditions in vigilant attention, concentration, or declarative memory. These results provide preliminary evidence supporting the incorporation of short-wavelength (blue) enriched, higher melEDI lighting in the built environment to facilitate learning and task performance in young adults following modest sleep restriction.

To our knowledge, this is the first study in moderately sleep-restricted young healthy adults to find a robust improvement in procedural learning on a motor task, both in performance and accuracy, induced by higher melEDI light exposure during the day. Consistent with our findings, high melEDI light exposure (6500K) during the evening improved procedural learning in older adults (mean age > 60 years) who had UV-blocking (0% light transmission between 300 and 360 nm) intraocular lens (IOL) replacement compared to older adults with blue-blocking IOLs (0% of light transmission between 300 and 400 nm and ~50% transmission between 410 and 480 nm) (21). Together, these results demonstrate that short-wavelength light exposure facilitates procedural learning, suggesting that the melanopic system is mediating the direct effects of light on learning, as has been shown previously for other cognitive domains [e.g., vigilance (3, 5, 14)].

Working memory and cognitive processing speed, as assessed by the Addition Task (26), was also better under higher melEDI light, exhibiting a clear linear dose-response relationship with melanopic illuminance. These results are consistent with previous studies showing improved working memory during exposure to short-wavelength enriched light (9, 33–35). Studies in similar age groups, but with shorter duration (≤ 30 min) and monochromatic exposures have also shown that short-wavelength light exposure activates the brain regions associated with working memory (35), including the prefrontal cortex [PFC (33)] whose activation is positively correlated with processing speed and accuracy on a working memory task. Our results extend these findings to show observed improvement in working memory and cognitive processing speed with short-wavelength light exposure in individuals following moderate sleep restriction, and under naturalistic long-duration exposures during the day.

Importantly, our results show that not all cognitive domains are responsive to short-wavelength enriched light to the same extent. In the current study, improvements were not observed in tests of vigilance and reaction time in these modestly sleep-restricted participants. While these findings are in contrast to previous reports of positive effects of short wavelength-enriched light exposure on sustained attention (3, 4, 9–13, 36), not all studies have shown positive effects, especially during daytime exposures (17, 37–40). The inconsistent findings may be due to differences in exposure characteristics including exposure duration and timing, and differences in spectra and intensity between experimental groups. Moreover, other factors such as prior sleep deficiency (40), light history (36), and pupillary constriction due to differences in light spectra and subsequently differences in retinal exposure (8) may have contributed to the differences in performance observed in our study compared to prior studies, especially given that the effects of light exposure on performance during the day are smaller compared to exposure at night (14). Despite these differences, our results are internally consistent in that the two associated cognitive domains, namely sustained attention and concentration (PVT and d-2 tasks, respectively) were not different between lighting conditions. Importantly, while short wavelength-enriched light exposure did not improve all cognitive domains that were assessed, there was no evidence that light with lower melanopic illuminance was better. Future studies with higher statistical power are necessary to better understand the underlying relative photoreceptor contributions affecting different cognitive domains.

Interestingly, the subjective ratings of sleepiness in response to light were not consistent between different scales. Although we found that generally, higher melanopic illuminance was associated with less subjective sleepiness assessed by the KSS, which is consistent with some previous studies (2–4, 15, 34, 36), we did not see an effect of lighting condition on the self-rated sleepy-alert VAS. This may suggest that the different tests have differential sensitivity for detecting the effects of light on self-reported outcomes and may help to understand inconsistencies in the effect of light on subjective sleepiness and alertness between studies (8). Moreover, the inconsistency between subjective ratings of sleepiness and alertness and objective performance is in agreement with prior reports showing that subjectively reported sleepiness is often an unreliable indicator of objectively assessed neurobehavioral performance (40, 41). Surprisingly, subjective KSS sleepiness ratings did not increase with lower melEDI but was in fact highest under the highest melEDI exposure. This unexpected finding suggests that overall spectral composition of the light, besides only melanopic illuminance, may be influencing some neurobehavioral responses.

Our current study has several limitations. Given that the differences in spectra and melanopic illuminance between the light conditions may not have been large enough to differentiate their effects in some performance domains (e.g., sustained attention) and we only examined the effects of a single intensity, further studies are required to better test a broader range of melanopic illuminances and spectra. Similarly, future work is needed to better evaluate the time-course of light effects on performance. For example, declarative memory was assessed only once in the current study, several hours into the LE, although previous studies have shown a positive effect of light when declarative memory was assessed much sooner after light onset (9, 20). Furthermore, administering the word pairs task for the first time after lights on would have allowed us to assess the effects of light not only on recall but also on learning. Finally, future work is needed to examine the chronic (multiple days) vs. acute (single day) effects of light on performance, including the effects of light on sleep and the impact that this has on subsequent performance, for example sleep dependent learning and memory (29, 30).

The acute alerting response to light may be an effective non-invasive intervention for preventing the neurobehavioral performance impairment associated with inadequate sleep. Sleep restriction negatively influences many aspects of cognitive performance and mood (41, 42), even when only restricted to 7 h of sleep per night (43), as in the current study where participants could only sleep at most for 7 h given that their time in bed was fixed at 7 h. Furthermore, restricted and irregular sleep also impairs performance to a greater extent than stable sleep loss (40) and has also been shown to affect GPA in college students (23, 44), a population with a high prevalence of insufficient sleep. For example, in a study of college students (*n* = 1,125), more than 60% were categorized as poor-quality sleepers and a quarter reported getting <6.5 h of sleep per night (22). A sleep duration <7 h in college-aged adults is likely insufficient as when young adults are given an extended sleep opportunity (16 h per night for 9 nights) their total sleep duration has been shown to approach an asymptote of 8.7 h per night (45). Based on the findings of the current study, the acute alerting effects of light may be a useful countermeasure for performance impairment associated with sleep deficiency in this population; however, additional studies are required to evaluate the impact of sleep regularity and varying extents of sleep restriction (e.g., 5 vs. 7 h per night) on the acute alerting effects of light exposure. Blue-enriched light has been tested as an alertness countermeasure in school and college students and showed improvements in processing speed, concentration, and reading speed [e.g., (28, 46, 47)]. Similar benefits of short-wavelength enriched light exposure on performance and alertness have also been observed in office settings (31, 48). These studies, coupled with our results showing that short wavelength enriched long-duration light exposure during the daytime improves working memory/processing speed, procedural learning and subjective sleepiness, support the incorporation of short wavelength enriched white light in indoor environments to enhance learning and cognitive performance.

## Data Availability Statement

The raw data supporting the conclusions of this article will be made available by the authors, without undue reservation.

## Ethics Statement

The studies involving human participants were reviewed and approved by Partners Human Research Committee. The patients/participants provided their written informed consent to participate in this study.

## Author Contributions

SL and SR contributed to the initial concept of the study. LG, RS, SL, and SR contributed to the design of the study. LG, BK, MM, and SR contributed to, or oversaw, participant recruitment, and data collection. LG, RS, and SR contributed to the analysis of the data. All authors contributed to the interpretation of the data and drafting of the manuscript. All authors have contributed to and approved this manuscript.

## Conflict of Interest

SL reports commercial interests from the last 3 years (2017–2020) unrelated to the study reported herein but are reported in the interests of full disclosure. SL has received consulting fees from the BHP Billiton, EyeJust Inc., Noble Insights, and Team C Racing; honoraria and/or paid travel from BHP Billiton, DIN, Emory University, IES, Ineos, MIT, Roxbury Latin School, SLTBR, Solemma and Teague; has current consulting contracts with Akili Interactive; Apex 2100 Ltd.; Consumer Sleep Solutions; Headwaters Inc.; Hintsa Performance AG; Light Cognitive; Lighting Science Group Corporation; Mental Workout; PlanLED; Six Senses; Stantec; and Wyle Integrated Science and Engineering; has received unrestricted equipment gifts from Bionetics Corporation and F. Lux Software LLC; royalties from Oxford University Press; and has served as a paid expert in legal proceedings related to light, sleep, and health. He is an unpaid Board Member of the Midwest Lighting Institute (non-profit) and was the Program Leader for “Safety and Productivity Improvements” in the CRC for Alertness, Safety, and Productivity from 2015 to 2019. Dr. Lockley's interests were reviewed and managed by Brigham and Women's Hospital and Partners HealthCare in accordance with their conflict of interest policies. SR holds patents for Prevention of Circadian Rhythm Disruption by Using Optical Filters and Improving sleep performance in subject exposed to light at night; SR owns equity in Melcort Inc.; has provided paid consulting services to Sultan and Knight Limited, Bambu Vault LLC, Lucidity Lighting Inc.; and has received honoraria as an invited speaker and travel funds from Starry Skies Lake Superior, University of Minnesota Medical School, PennWell Corp., and Seoul Semiconductor Co., Ltd. These interests were reviewed and managed by Brigham and Women's Hospital and Partners HealthCare in accordance with their conflict of interest policies. The remaining authors declare that the research was conducted in the absence of any commercial or financial relationships that could be construed as a potential conflict of interest.
