# Supplementary material for: Daytime Exposure to Short Wavelength-Enriched Light Improves Cognitive Performance in Sleep-Restricted College-Aged Adults
Source: Front Neurol. 2021 Feb 22;12:624217. doi: 10.3389/fneur.2021.624217 (PMC7937889; doi:10.3389/fneur.2021.624217)
Supplement: Supplementary file 1 [file Data_Sheet_1.pdf]

Supplemental material to Grant et al.,

Daytime exposure to short wavelength-enriched light improves cognitive performance in sleep-restricted college-aged adults

Leilah K. Grant,[1,2] Brianne A. Kent,[1,2] Matthew D. Mayer,[1] Robert Stickgold,[3,4] Steven W. Lockley,[1,2] Shadab A. Rahman[1,2]

1. Division of Sleep and Circadian Disorders, Departments of Medicine and Neurology, Brigham and Women's Hospital, Boston, MA 02115, USA
2. Division of Sleep Medicine, Harvard Medical School, Boston, MA 02115, USA
3. Department of Psychiatry, Beth Israel Deaconess Medical Center, Boston, MA 02115, USA
4. Department of Psychiatry, Harvard Medical School, Boston, MA 021115, USA

Supplemental Materials and methods

Light exposure

During the LE, the monitor used to administer the neurobehavioral testing was calibrated to have an illumination of <2 lux at ~30 cm measured in the vertical plane from the center of the screen. The light exposure was delivered via two desk lamps fitted with LED chips that were placed on either side of the computer monitor on the testing station (Figure 1D). The experimental setup and light geometry were consistent throughout the LE and across participants. All luminaires were identical in dimension and color and no special markings were visible to participants that would differentiate the units. The lamps were turned on manually by investigators at preset times based on each individuals' inpatient sleep-wake routines (i.e., 2 h after habitual wake; Figure 1A). All lamps were turned on ~2 min prior to the scheduled start time of the LE, before the overhead room lights were turned off, to avoid exposure to darkness and dark adaptation of the photoreceptors prior to the start of the LE.

Sleepiness, performance and learning assessments

<u>Psychomotor Vigilance Task (PVT).</u> Following a baseline assessment prior to the start of LE, sustained attention was assessed hourly throughout the LE using the 10-min PVT. During the PVT a visual stimulus was presented at random intervals (1-9 sec) and the participant responded to the stimulus by pressing a button as soon as possible after seeing the stimulus. Mean reaction time and the number of attentional failures (reaction times > 500ms) were calculated for each PVT session.

<u>Addition Task.</u> A 2-min mental Addition Task was used as a measure of working memory and processing speed and was administered hourly throughout the LE. Baseline performance was assessed prior to the start of the LE. During this task, participants were required to add together as many pairs of 2-digit numbers as possible within a 2-min interval. The main outcome measures included the number of attempted additions and the percentage of correct additions relative to the number of attempted additions.

d-2 test. Concentration was assessed using the d-2 test which was administered ~1 h before lights on and again 7 hours later (i.e., 6 h into the LE). The test consisted of the letters 'd' and 'p' with 1 to 4 dashes arranged above or below each letter, either individually or in pairs across 14 test lines. Participants were asked to scan each test line and cross out as many d's with two dashes within a 20-s interval per line. Main outcomes from the d-2 task included: (1) concentration performance, which was calculated by subtracting errors of commission (i.e., crossing out any "p" or a "d" with fewer or more than two dashes) from the number of correctly processed items, and (2) percentage of errors, which was calculated as the proportion of total errors (errors of commission and omission) made relative to the total number of items processed.

Word Pairs. Declarative memory was assessed using the word pairs task during which participants learned a list of 40 word-pair associates ~1 hour before the light exposure began and recalled them 7 hours later (i.e., 6 hours into the LE). During the learning session, participants had a maximum of 5 trials prior to a final free recall test. For the learning trials, participants were presented with one word of each word pair and asked to type in the other word with feedback given on whether the typed word was correct or not. If participants met learning criterion (20 correct words pairs, i.e., 50%) prior to the 5th trial, the learning trials stopped, and the participants were immediately asked to complete a final free recall. During free recall, participants were presented with the first word from each word pair and asked to type the second word with no feedback given. Seven hours later, during the sixth hour of the LE, participants completed another free recall session where they were asked to type the word that went with each word that was presented. The main outcome measure was the percentage of words recalled correctly during the recall session during LE relative to the total number of correct word pairs recalled during the free recall immediately after the learning session.

Motor Sequence Task (MST). The MST was used to assess procedural learning, which was administered 30 min following the start of the LE and again 7 h later (~7.5 h from the start of the LE). The task consisted of 12 30-sec trials with 30-sec rest intervals between trials. In each trial, participants typed a 5-digit sequence as quickly and accurately as possible using a small keypad placed under their left hand. The sequences were 4-1-3-2-4 and 1-4-2-3-1 in session 1 and session 2, respectively. The main outcome measures were improvements in performance speed and accuracy, which were calculated as the percentage change in the number of correct sequences and the number of errors (higher accuracy value represent poorer performance), respectively, between the first trial and the average of the last three trials (trials 10, 11 and 12).

Karolinska Sleepiness Scale (KSS). Subjective sleepiness was rated hourly throughout the LE using the KSS, a 9-point scale from 1-"very alert" to 9-"very sleepy, fighting sleep."

General health and wellbeing scales. Participants rated their general health and wellbeing on a 7-item assessment using a 100 mm bipolar visual analogue scale (VAS) for each pair of scale items presented. Participants were asked to place a mark on the line to represent their current state along the continuum. The 8 VAS items included 1) Sleepy – Alert, 2) Calm – Stressed, 3) Sad – Happy, 4) Healthy – Sick, 5) Energetic – Physically Exhausted, 6) Mentally Exhausted

– Sharp, 7) Tired to Death – Fresh as a Daisy, and 8) Motivated – Unmotivated. The VAS were administered hourly during the LE.

Headache and Eye Strain Scale. A 7-item scale was used to assess light comfort during the first and the seventh hour of the LE. The scales evaluated irritability, headache, eye strain, eye discomfort, eye fatigue, and blurred vision, which were rated from 0 – “absent” to 4 – “severe”.

Supplemental Table 1. Spectral characteristics of ambient fluorescent light settings *

Lighting condition	Radiometric and photometric values (380-780 inclusive)		Retinal photopigment Weighted Illuminances (α-opic EDI lux)					DER
	Irradiance μW/cm^2	Photopic illuminance Lux	S cone	Melanopsin	Rod	M cone	L cone	Melanopsin
4100K ambient <15 lux	2.73	8.79	4.06	7.80	8.82	6.24	5.78	0.66
4100K ambient 150 lux	47.49	157.31	72.87	137.92	157.38	106.74	95.83	0.61

* α-opic EDI and DER values for each light source were derived from the CIE S 026:2018 Toolbox V1.049. Spectral measurements were taken in the horizontal plane at a height of 182cm. EDI = equivalent daylight (D65) illuminance; DER = daylight (D65) efficacy ratio.

Supplemental Table 2. Mean ± SEM and ANOVA results for baseline test outcomes by light exposure condition*

Test outcome	Conventional low-melEDI	Daylight-like low-melEDI	Conventional high-melEDI	Daylight-like high-melEDI	F (p-value)
	M (SEM)	M (SEM)	M (SEM)	M (SEM)	
Objective measures					
PVT reaction time (ms)	280.40 (9.83)	268.40 (7.82)	283.40 (16.08)	287.70 (14.70)	0.43 (0.73)
PVT attentional failures	2.89 (0.70)	1.00 (0.53)	1.70 (0.53)	2.67 (1.24)	4.19† (0.24)
Additions % correct	90.60 (1.53)	94.18 (1.74)	92.11 (2.72)	95.24 (2.08)	3.78† (0.29)
Additions # attempted	23.56 (4.29)	24.89 (3.71)	20.10 (2.36)	20.89 (3.22)	1.67† (0.76)
d-2 CP	226.30 (15.49)	241.50 (16.94)	230.50 (10.98)	227.10 (13.81)	0.22 (0.88)
d-2 % errors	2.93 (0.76)	3.07 (0.67)	3.29 (0.47)	3.74 (0.73)	1.62† (0.66)
Subjective measures					
KSS	4.33 (0.65)	3.67 (0.60)	3.40 (0.50)	4.11 (0.61)	1.53† (0.68)
Sleepy – Alert	65.20 (6.72)	79.12 (7.89)	70.74 (7.87)	69.46 (9.14)	2.90† (0.41)
Calm – Stressed	27.60 (6.09)	20.72 (4.99)	21.04 (4.8)	16.90 (6.72	2.12† (0.57)
Sad – Happy	77.12 (5.36)	78.44 (5.51)	84.05 (4.82)	75.76 (5.70)	0.49 (0.69)
Healthy – Sick	19.77 (3.14)	12.64 (4.24)	12.70 (3.73)	25.35 (9.42)	3.95† (0.27)
Energetic – Exhausted	50.02 (3.62)	36.34 (7.30)	27.98 (7.38)	39.76 (7.16)	1.93 (0.14)
Exhausted – Sharp	63.08 (3.60)	69.80 (6.21)	76.59 (6.37)	69.61 (6.92)	4.43† (0.22)
Tired – Fresh	60.96 (3.66)	73.22 (6.13)	75.92 (5.58)	65.37 (6.63)	1.53 (0.23)
Motivated – Unmotivated	30.46 (4.11)	23.21 (7.71)	19.75 (5.65)	15.80 (4.26)	1.20 (0.33)

* M=mean; N = the number of participants in each group that were included in the analysis following removal of outliers; † denotes Kruskal-Wallis statistic where data were not normally distributed.

Supplemental Table 3. The number of participants in each condition reporting either None/Mild or Moderate/Severe symptoms for each Headache and Eye Strain Scale item

Headache and Eye Strain Scale Item	Symptom severity	Conventional low-melEDI	Daylight-like low-melEDI	Conventional high-melEDI	Daylight-like high-melEDI
SESSION 1 – Start of LE					
Irritability	None/Mild	9	8	9	9
	Moderate/Severe	0	0	1	0
Headache	None/Mild	9	8	10	9
	Moderate/Severe	0	0	0	0
Eye Strain	None/Mild	8	8	9	8
	Moderate/Severe	1	0	1	1
Eye Discomfort	None/Mild	8	8	9	7
	Moderate/Severe	1	0	1	2
Eye Fatigue	None/Mild	8	8	9	8
	Moderate/Severe	1	0	1	1
Blurred Vision	None/Mild	9	8	9	9
	Moderate/Severe	0	0	1	0
SESSION 2 – End of LE					
Irritability	None/Mild	8	7	9	8
	Moderate/Severe	1	1	1	1
Headache	None/Mild	9	8	10	7
	Moderate/Severe	0	0	0	1
Eye Strain	None/Mild	8	7	9	5
	Moderate/Severe	1	1	1	3
Eye Discomfort	None/Mild	8	7	9	8
	Moderate/Severe	1	1	1	1
Eye Fatigue	None/Mild	7	7	9	8
	Moderate/Severe	2	1	1	1
Blurred Vision	None/Mild	9	8	9	9
	Moderate/Severe	0	0	1	0
